# Molecular Dynamics Simulation of Helium Barrier Performance of Modified Polyamide 6 Lining of IV Hydrogen Storage Tank with Montmorillonite

**DOI:** 10.3390/molecules28083333

**Published:** 2023-04-10

**Authors:** Ping Wu, Jiaming Zhang, Zhenhan Yang, Jianping Zhao

**Affiliations:** 1 School of Mechanical and Power Engineering, Nanjing Tech University, Nanjing 211816, China; 2 Institute of Reliability Centered Manufacturing (IRCM), Nanjing Tech University, Nanjing 211816, China

**Keywords:** molecular dynamics, polyamide 6, hydrogen permeability

## Abstract

In order to investigate the type IV hydrogen storage bottle with better hydrogen storage capacity, the polymer lining of the hydrogen storage bottle was further developed. In this paper, the molecular dynamics method was used to simulate the helium adsorption and diffusion processes within a modified montmorillonite (OMMT)-filled polyamide 6 (PA6) system. The effects of the barrier properties of the composites were investigated at different filler contents (3%, 4%, 5%, 6% and 7%), different temperatures (288 K and 328 K) and different pressures (0.1 MPa, 41.6 MPa, 52 MPa and 60 MPa) for certain contents. It was found that when the filler content was 5%, the permeability coefficient of the material was lower than 2 × 10^−13^ cm^3^∙cm/(cm^2^∙s∙Pa) and the barrier performance was the best. The modified filler with 5% OMMT/PA6 at 328 K still had the strongest barrier performance. When the pressure increased, the permeability coefficient of the modified material first decreased and then increased. In addition to this, the effect of the fractional free volume on the barrier properties of the materials was also investigated. This study provides a basis and reference for the selection and preparation of polymer linings for high-barrier hydrogen storage cylinders.

## 1. Introduction

Nowadays, the production and application of hydrogen have quite mature technology, but the storage of hydrogen is the main difficulty that restricts the application and promotion of hydrogen energy [1,2,3]. High-pressure hydrogen storage has the advantages of a simple equipment structure and fast charging and discharging speeds, and it is the main movable hydrogen storage method at present [4,5,6]. The development of a fully composite hydrogen storage bottle with a polymer lining is the direction of development of high-pressure hydrogen storage containers today [7,8]. Due to the flammable and explosive nature of hydrogen, many scholars have used small-molecule gases (He) for alternative treatment in the study of hydrogen leakage and have linked helium to hydrogen diffusion in order to convert the two more reliably [9,10,11], so the study of how to improve the small-molecule gas barrier properties of plastic liners has become a key issue. Some studies have shown that polyethylene has good gas barrier properties and can be used as a plastic liner for hydrogen storage cylinders [12]. However, compared with polyethylene, polyamide is more suitable as a plastic lining for hydrogen storage bottles due to its strong molecular polarity and low gas permeability, and many scholars have conducted research in this area [13,14].

Humpenoder [15] studied the permeability of helium, hydrogen and methane in PA in the temperature range of 0–25 °C, and the permeability of all gases decreased with increasing temperature. Pepin et al. [16] showed that at a temperature of 55 °C and a hydrogen pressure of 18 MPa, PA12 dissolved more hydrogen than PA6, but the gas diffused more rapidly in PA12 than in PA6. To further investigate the gas barrier properties of polyamide, Lafitte et al. [17] prepared a series of PA11/PHAE blends by melt blending and found that different ratios had a significant effect on the properties of the blends, and the variation in hydrogen barrier properties was related to the composition of the blends, which increased with the increase in PHAE content in the blends.

With the rise of nanocomposites, scholars have attempted to prepare nanocomposites by incorporating inorganic nano-fillers into polymer matrices [18]. The incorporation of inorganic nano-fillers can achieve better properties compared to ordinary composites [19,20]. Fasihii [21] improved the performance of polyamide-6 (PA6) nanocomposite films by adding different concentrations of nano-clay to PA6. It was found that there was a significant decrease in the permeability and diffusivity of the nanocomposite membranes as the nano-clay content increased. Montmorillonite is widely used as a nano-filler in polymer composites due to its stiffness, strength, structural stability and exceptional ion-exchange and dispersion properties [22,23,24]. However, the structure of montmorillonite and its inorganic components determine the surface to be hydrophilic, but most polymeric matrices are hydrophobic, so there is an incompatibility between montmorillonite and the polymeric matrix. In addition, the strong electrostatic interactions that occur between the exchangeable cations of the montmorillonite layers make them tightly bound and difficult to disperse in the polymer matrix. Therefore, suitable coupling agents need to be selected to modify the montmorillonite by replacing the exchangeable cations in the montmorillonite with suitable long-chain cations. The compatibility of the modified montmorillonite with the polymer matrix and the bonding at the interface are greatly increased [25,26]. Usually, the coupling agent chosen is quaternary ammonium salt or phosphorus salt [27], with octadecyl trimethylammonium chloride [28] being a commonly used quaternary ammonium salt modified coupling agent. Suman Kumar [29] combined silane-modified montmorillonite with LDPE and found that the oxygen transmission rate of the composite was reduced by one-third with the addition of modified montmorillonite compared to pure LDPE. In another study, Moises [30] investigated the permeability of oxygen in PP nanocomposites with added montmorillonite, which decreased linearly with the increasing weight percentage of the added montmorillonite. In summary, the addition of montmorillonite changes the permeability of the material.

In this paper, a molecular model of nanocomposites with polyamide 6 as the polymer substrate and modified montmorillonite as the nano-filler was constructed by means of a molecular dynamics simulation. Due to the high difficulty factor of hydrogen experiments and tests, simulations with helium instead of hydrogen can more simply reflect the gas barrier properties of the materials. By studying the diffusion coefficient of helium molecules in the polymer composite model and investigating the effect of different filler ratios on the gas permeation of the nanocomposites under different temperature and pressure conditions, better barrier performance materials were obtained.

## 2. Simulation

### 2.1. Modified Montmorillonite (OMMT)/Polyamide-6 (PA6) Model Construction

**Figure 1:**
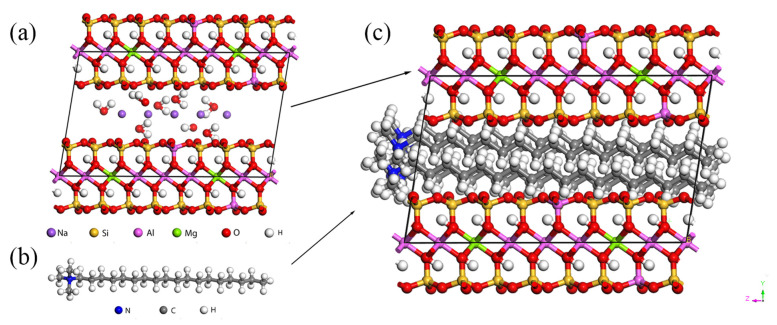
Molecular Models: (**a**) original model of MMT, (**b**) octadecyl trimethylammonium chloride, and (**c**) montmorillonite model after surface modification.

Montmorillonite is an aluminosilicate mineral with two silica-oxygen tetrahedral layers interspersed with an aluminum-oxygen octahedral layer, which has a strong ion-exchange capacity [31]. MMT cells with lattice sizes of a = 5.23 Å, b = 9.06 Å and c = 12.5 Å were constructed using Material Studio. Atoms were added according to their spatial coordinates and the interlayer cation coordinates from Table 1, and then a supercell of size 4a × 2b × 1c was built. The SPC/E model was chosen for the potential energy model of water in the montmorillonite-water-ion system, where water is a rigid molecule [32,33,34]. The charges and Lennard-Jones parameters of each atom in the initial model are shown in Table 1. The MMT model was obtained according to the substitution principle, and the MMT interlayer cations were six Na^+^. Since MMT is hydrophilic, the surface modification of the six interlayer cations of MMT was replaced with octadecyl trimethylammonium chloride for better compatibility with the polyamide materials. The obtained model is shown in Figure 1.

**Figure 2:**
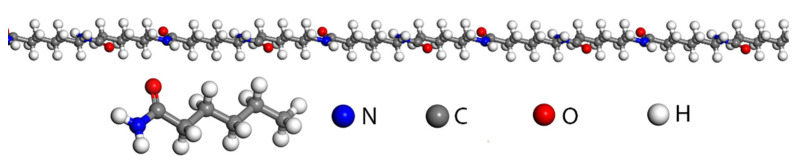
One hundred repeating units of PA6 molecules.

**Figure 3:**
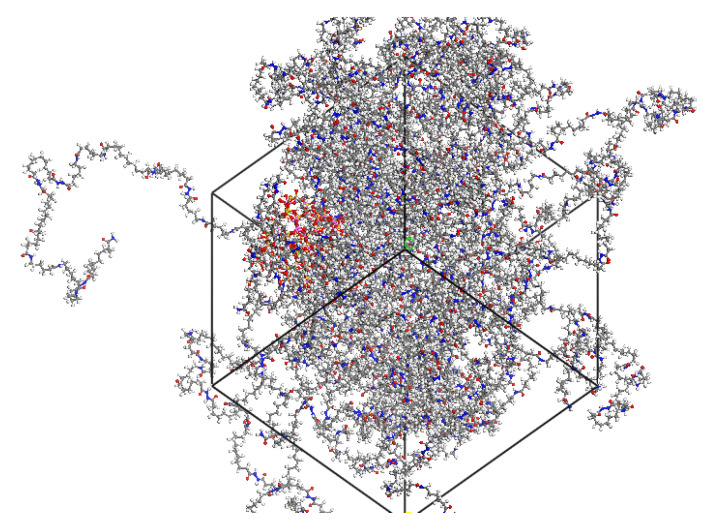
Composite model of PA6 single chains and MMT surface modification.

One hundred repeating units were established to form random single chains of PA6 molecules (Figure 2), and then the composite models of PA6 single chains and MMT surface modification were constructed by using the random copolymer option, as shown in Figure 3. In the composite model system, a single modified MMT molecule was first added, and then the mass fraction of OMMT was changed by gradually increasing the number of PA6 single chains. The mass fractions of OMMT were 3%, 4%, 5%, 6% and 7%. The unit cell sizes for different mass fractions were approximately 75.51 Å, 68.84 Å, 64.11 Å, 60.53 Å and 56.47 Å, respectively, and the lattice sizes in three directions (a, b and c) were the same for all the cell units.

### 2.2. Simulation of the Calculation Process

In order to achieve the most stable initial state of the established model, the model was first structurally optimized and energy-minimized by the geometric optimization method with a maximum number of iterative steps of 5 × 10^4^. Next, the composite system was cyclically annealed using Anneal. For a more rational molecular structure, the annealing cycle was set to 10 times with an initial temperature of 300 K, and the mid-point of each annealing cycle was 500 K (this was optimized after each cycle to make the material configuration more reasonable). Molecular dynamics (MD) was then used to extract the optimized model with the minimum energy for kinetic simulation. The thermodynamic ensemble of NPT was selected under the UFF force field, and the kinetic optimization was performed for 1000 ps at 298 K and 0.1 MPa. The Andersen heat bath method was selected for temperature control and the Berenson pressure bath method for pressure control.

**Figure 4:**
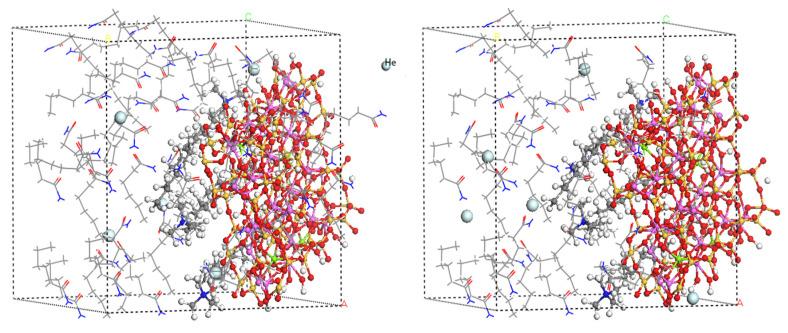
Schematic diagram of unit cell model with helium and modified montmorillonite. The helium molecules and modified montmorillonite are represented in space-filling models, and PA6 is represented in wireframe models.

The solubility coefficients of helium were obtained using adsorption isotherms at pressure variations ranging from 0.01 kPa to 10,000 kPa. The working condition of a type IV hydrogen storage tank is at 52 MPa with a safety pressure of the storage tank of 60 MPa. The operating pressure of the IV hydrogen storage tank is generally 41.6 MPa, which is 0.8 times the working pressure. Therefore, four different pressures (0.1 MPa, 41.6 MPa, 52 MPa and 60 MPa) were simulated by using sorption. In order to obtain the permeation coefficient, helium permeation was performed using fixed pressure on the configuration. Then, an NPT dynamics simulation of 500 ps under NPT for a system containing helium molecules was conducted, and a schematic diagram of the unit cell model is shown in Figure 4. The analysis function was used to calculate the mean square displacement curve (MSD) of the gas molecules to obtain the diffusion coefficient of helium.

The Universal force field (UFF) [35] was used throughout the simulation. For non-bonded interactions, van der Waals interaction was performed using the atom-based method and the Ewald method for electrostatic interactions.

The solubility coefficients of gas molecules can be obtained by fitting isothermal adsorption curves to obtain adsorption equations and then performing concentration conversion [36]:

(1) $$C=k_{D}P+C_{H}\frac{bP}{1+bP}$$

(2) $$S=\underset{p\to 0}{\lim}\frac{C}{P}=k_{D}+C_{H}b$$

where $k_{D}$ is the Henry constant, $C_{H}$ is the Langmuir capacity parameter, *b* is the Langmuir parameter, *P* is the pressure and *C* is the adsorption of gas molecules.

The diffusion coefficient of a gas molecule is an equation relating velocity and time, and the helium diffusion coefficient can be calculated using the following equation [37,38]:

(3) $$D=\frac{1}{6N}\underset{t\to \infty }{\lim}\sum_{i=1}^{N}{\left[{\vec{r}}_{i}\left(t\right)-{\vec{r}}_{i}\left(0\right)\right]}^{2}$$

where *D* is the gas diffusion coefficient, *N* is the number of helium atoms, ${\vec{r}}_{i}\left(t\right)$ and ${\vec{r}}_{i}\left(0\right)$ are the position vectors of the *i* helium atom at moment *t* and moment 0, respectively, and $\frac{1}{N}\sum_{i=1}^{N}{\left[{\vec{r}}_{i}\left(t\right)-{\vec{r}}_{i}\left(0\right)\right]}^{2}$ represents the mean square displacement of the gas molecules.

The permeability of gas molecules in the composite model can be characterized by calculating the solubility and diffusion coefficients of gas molecules:

(4) P=D×S

where *P* is the permeability coefficient and *D* is the gas diffusion coefficient.

In order to obtain the fractional free volume (FFV) of the system, the system model was analyzed using the Atom Volumes and Surface tool. The Connolly radius was set to 1.40 Å based on the known van der Waals radius of the helium atom, and the Connolly surface of the system was calculated to obtain the FFV of the system.

## 3. Results and Discussion

### 3.1. Influence of Filler Ratio on Permeability Coefficient

**Figure 5:**
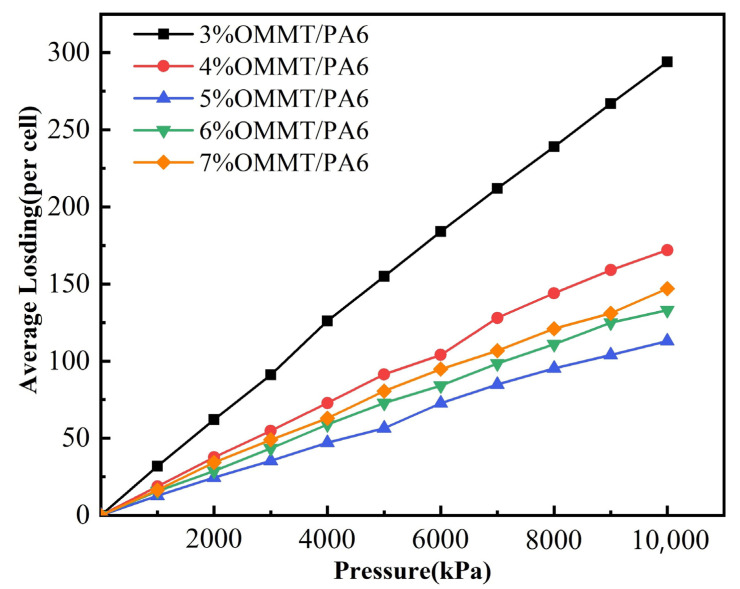
Isothermal adsorption curves of helium in different systems.

The results obtained from the simulations are shown in Figure 5. Figure 5 shows the isothermal adsorption curves of helium by OMMT/PA6 with different contents. From Figure 5, it can be seen that the average adsorption of helium in the composites kept a steady increase, but the gas adsorption of the composites gradually decreased with the increase in the OMMT content added to PA6. When approximately 5% of OMMT was added to PA6, the adsorption amount of helium reached its minimum. Then, with further increases in the OMMT content, the helium adsorption started to increase. This means that the addition of MMT affects the helium adsorption region. The adsorption of gases is correlated with the solubility of the material, and an increase in the OMMT content leads to a decrease and then an increase in the solubility of the material. At 5% OMMT/PA6, the solubility is the lowest, and the gas adsorbable area is the lowest.

**Figure 6:**
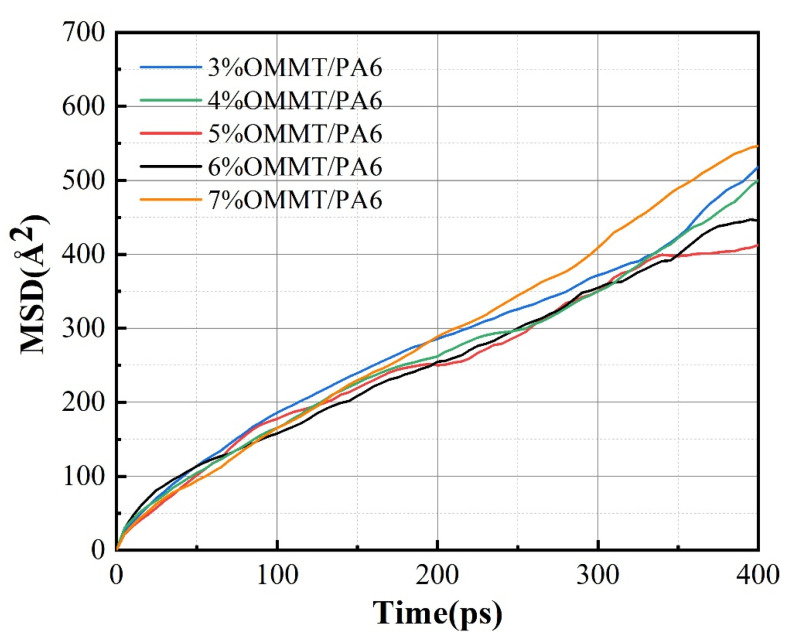
MSD curve of helium in different systems at 288 K and 0.1 Mpa.

**Figure 7:**
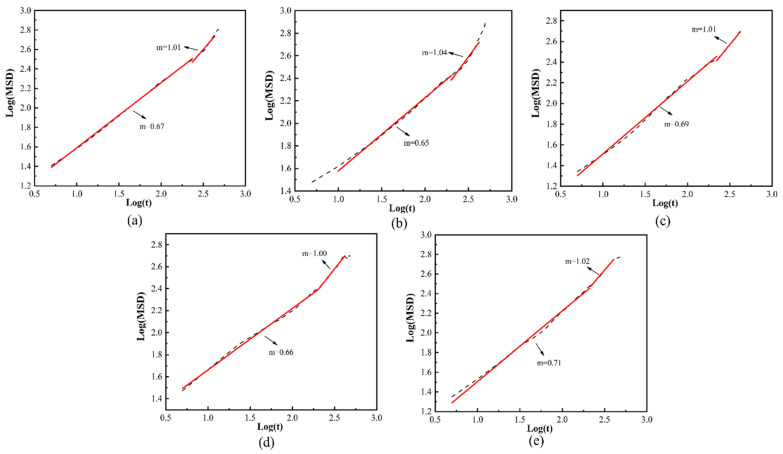
Logarithmic plot of MSD versus simulation time for helium in different systems at 288 K: (**a**) 3%, (**b**) 4%, (**c**) 5%, (**d**) 6% and (**e**) 7%.

From Figure 6, it can be seen that the MSD curve has a linear relationship with time. Before Einstein diffusion occurs, the system may experience abnormal diffusion due to structural reasons. In order to verify that the system has experienced normal diffusion, the diffusion characteristics need to be determined by:

(5) $${\langle \left[{\vec{r}}_{i}\left(t\right)-{\vec{r}}_{i}\left(0\right)\right]}^{2}\rangle \propto t^{m}$$

where m is neither 1 nor 2. It can be verified by checking the slope of the logarithmic curve of the mean square displacement and the time [39,40]. The slope of the curve for log(MSD)~log(*t*) is shown in Figure 7. The moment of occurrence of normal Einstein diffusion in all systems is approximately *t* = 220 ps, and the diffusion reaches a stable state.

The MSD curve was fitted to find the diffusion coefficient from Equation (3). The solubility coefficients, diffusion coefficients and permeability coefficients of the model calculated from the equations are shown in Table 2. From Table 2, it can be seen that the addition of OMMT has an effect on the solubility coefficient and diffusion coefficient of the material. With the increase in the OMMT content, the solubility coefficient and diffusion coefficient of the composites both decreased and then increased, which led to the decrease and then increase in the permeability coefficient of the material. When the OMMT content reached 5%, the solubility, diffusion and permeability coefficients of the material reached their minimums. Compared with other systems, the gas barrier of 5% OMMT/PA6 is as low as 2 × 10^−13^ cm^3^∙cm/(cm^2^∙s∙Pa), indicating that this system has the best gas barrier. Compared with the material with 3% OMMT content, the 5% OMMT content has approximately 60% lower solubility, 15% lower diffusion coefficient and 60% lower permeability coefficient. When raising the OMMT content to 6%, both solubility and diffusion coefficients increased slightly, and the permeability coefficient increased by about 30% compared to the system containing 5% OMMT. The solubility and diffusion coefficients continued to increase when the addition of OMMT reached 7%. The reason for these results may be that the addition of a small amount of OMMT breaks the channels for the diffusion of gas molecules in the PA6 system and increases the degree of complexity of the molecular diffusion channels. Due to the irregular spatial distribution of the PA6 molecular chains and montmorillonite molecules in the model, the increased channel complexity impedes the diffusive movement of the gas, resulting in a lack of sufficient energy for further movement of the gas within the system; therefore, the penetration of the gas molecules is limited. However, the addition of too much OMMT filler leads to more molecular diffusion channels in the system, so the permeation coefficient of the composite increases slightly. From this, it can be found that the addition of 5% OMMT content to PA6 is the most suitable in order to obtain the best barrier material.

### 3.2. Effect of Temperature on Permeability Coefficient

**Figure 8:**
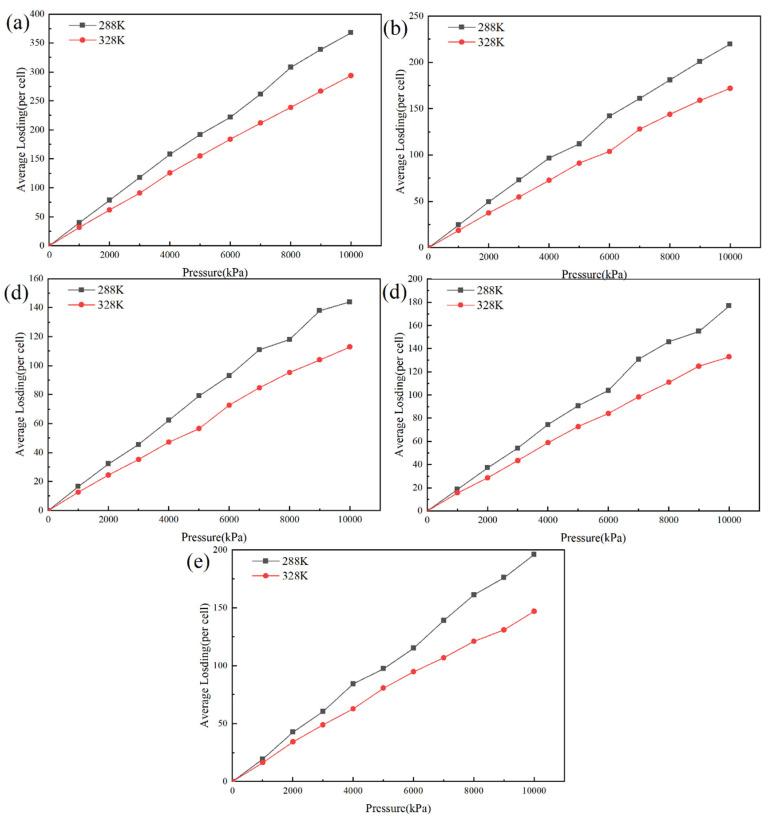
Isothermal adsorption curves of helium in different OMMT content systems at 328 K and 288 K: (**a**) 3%, (**b**) 4%, (**c**) 5%, (**d**) 6% and (**e**) 7%.

Figure 8 shows the isothermal adsorption curves of helium for different OMMT content systems, with the black and red lines indicating the solubility curves at 288 K and 328 K, respectively. It can be seen from the figure that the solubility coefficient of helium decreases with an increase in temperature. When the temperature increases to 328 K, the adsorption coefficients of the systems with different OMMT contents all decrease by approximately 20%. This takes place because the increase in temperature accelerates the movement of the molecules in the system and reduces the adsorption capacity of the system for gas molecules.

**Figure 9:**
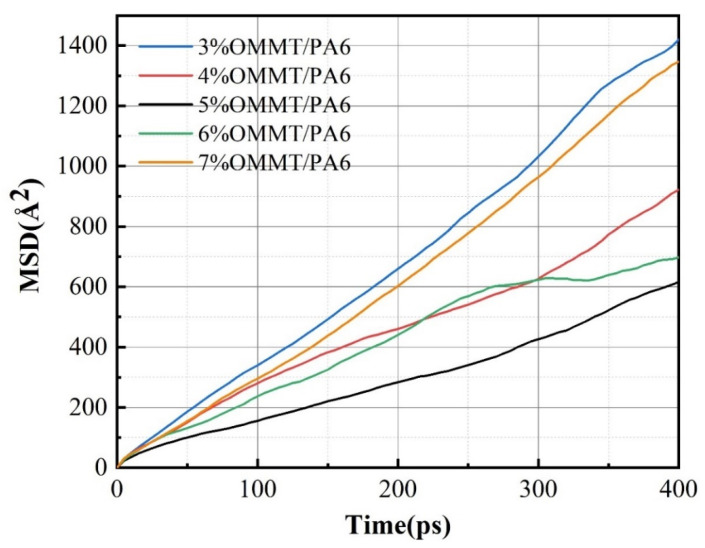
MSD curve of helium in different systems at 328 K.

**Figure 10:**
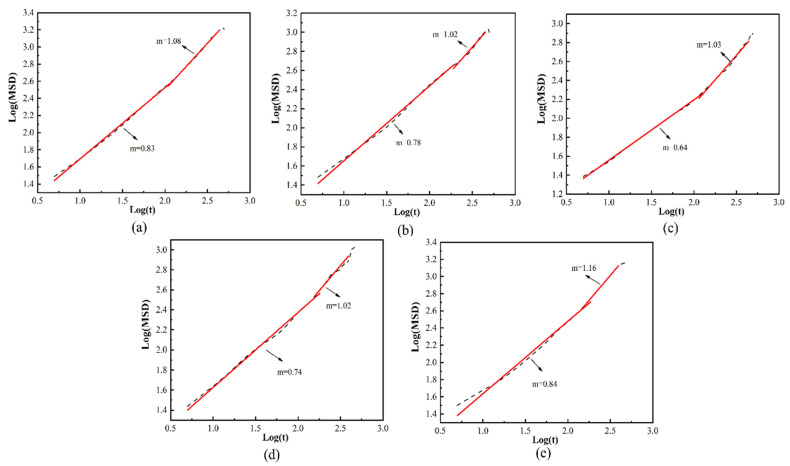
Logarithmic plot of MSD versus simulation time for helium in different systems at 328 K: (**a**) 3%, (**b**) 4%, (**c**) 5%, (**d**) 6% and (**e**) 7%.

Figure 9 shows the helium MSD curves for the systems with different OMMT contents at 328 K. From this, it can be seen that the increase in temperature leads to an increase in the slope of the absolute value of the MSD curve. The slope of the curve for log(MSD)~log(*t*) is shown in Figure 10. The moment of occurrence of normal Einstein diffusion in all systems is approximately *t* = 200 ps, and the diffusion reaches a stable state. The increase in temperature leads to a decrease in the time of the normal diffusion.

The solubility and diffusion coefficients at different temperatures are shown in Table 3. From the data in the table, it can be seen that the diffusion coefficients of OMMT/PA6 with different filler ratios increased compared to 288 K. Meanwhile, the permeability coefficient of OMMT/PA6 with a 3% filler ratio increased approximately 2.5 times to 10^−12^ cm^3^∙cm/(cm^2^∙s∙Pa) at 328 K compared to 288 K. The permeability coefficient of 4% OMMT/PA6 increased approximately 1.7 times, while the permeability coefficients of 5% OMMT/PA and 6% OMMT/PA increased by a factor of approximately 1.5. The permeability coefficient of 7% presented the highest increase in the system. It can be seen that temperature has a great influence on the permeability of the material, and there have been many studies that have demonstrated this phenomenon [41,42]. Furthermore, at 328 K, the permeability coefficients of the systems remained below 10^−12^ cm^3^∙cm/(cm^2^∙s∙Pa) except for the addition of 3% OMMT, which again indicates that the barrier properties of the materials were greatly improved by the addition of a certain content of OMMT. The diffusion and permeability coefficients of OMMT/PA6 increase with an increase in temperature because the thermal motion of helium molecules increases and the intermolecular collisions become stronger due to the increase in temperature.

### 3.3. Effect of Pressure on Permeability Coefficient

As can be seen in Figure 8, when the temperature is constant, the amount of helium adsorbed in the system is approximately proportional to the pressure, the solubility of the gas in the material hardly changes with pressure and the change in pressure has almost no effect on the change in the solubility coefficient and the permeability properties of the material. This takes place because the variation in pressure has little effect on the thermodynamic properties between the gas molecules and the modified polymer, and the solubility of the material remains essentially unchanged. Therefore, the solubility of the gas can be considered a constant value at different pressures.

**Figure 11:**
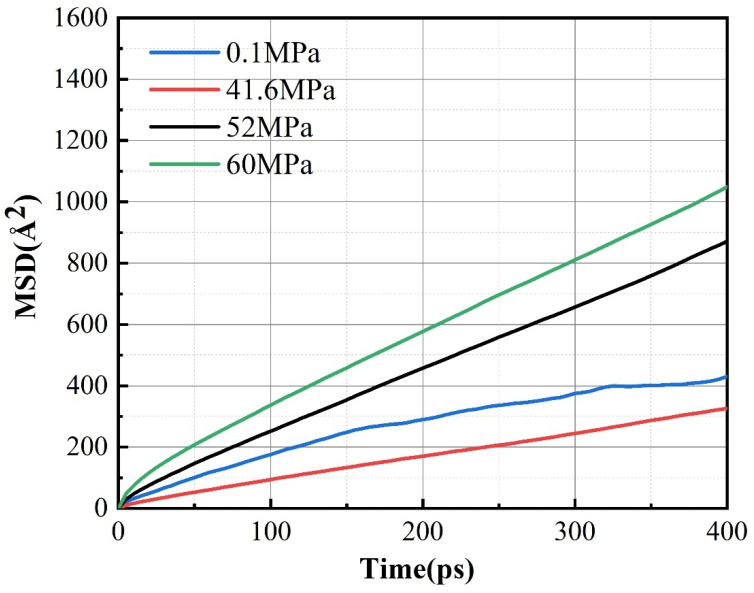
MSD curves for PA6 with 5% OMMT content of helium at different pressures at 288 K.

Figure 11 shows the MSD curves of the system with 5% OMMT content added at different pressures. It can be seen that the slope of the curve decreases and then increases with increasing pressure, and the pressure factor has an obvious effect on the diffusion characteristics of the modified material. The diffusion coefficients at different pressures obtained from the calculations are shown in Table 4. From the data in the table, it can be seen that the diffusion of helium molecules in the system is greatly affected. The diffusion coefficient of the material decreases slightly as pressure increases to 41.6 MPa. The type of gas molecule affects the permeability of the gas under pressure changes [43]. The permeability of soluble gases such as CO_2_ increases with increasing pressure, while the permeability of slightly soluble gases such as He decreases with increasing pressure. This has been demonstrated by the polymer hydrogen permeation experiments performed by Kanesugi [44]. The increase in pressure leads to a decrease in the permeability of helium and a decrease in the permeability coefficient. As pressure increases, the density of the material increases, which means that the intermolecular gaps are compressed, the volume of the material decreases and the molecules become tightly packed. The reduction of the molecular gaps reduces the free volume between molecules, which affects the diffusion channel of intermolecular gas and limits the diffusion of gas molecules. When pressure increases to 52 MPa and 60 MPa, the diffusion and permeability coefficients of the material increase. The increase in pressure provides kinetic energy for molecular motion. As pressure continues to increase, the effect of increasing the density of the material diminishes, although the material is compressed under pressure. This high pressure provides help for the molecular motion, greatly promoting the migration of gas molecules in the interior of the material; hence, pressure promotes the diffusion of helium molecules. In summary, pressure has a very strong influence on the gas barrier performance of the material.

### 3.4. Analysis of the Effect of Free Volume

**Figure 12:**
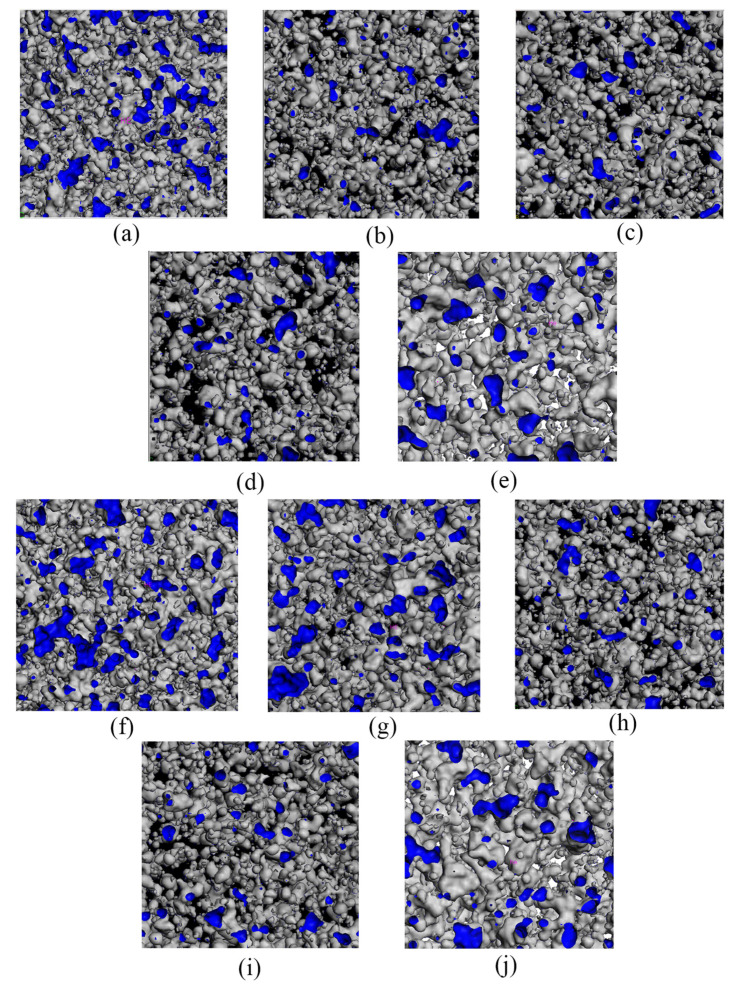
FFV of PA6 material with different OMMT contents at 288 K: (**a**) 3%, (**b**) 4%, (**c**) 5%, (**d**) 6% and (**e**) 7%; FFV of PA6 material with different OMMT contents at 328 K: (**f**) 3%, (**g**) 4%, (**h**) 5%, (**i**) 6% and (**j**) 7%.

**Figure 13:**
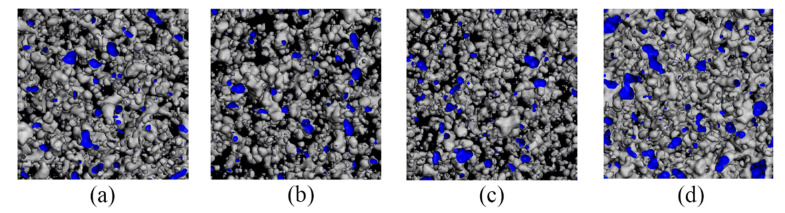
FFV of 5% OMMT/PA6 material at different pressures: (**a**) 0.1 MPa, (**b**) 41.6 MPa, (**c**) 52 MPa and (**d**) 60 MPa.

The internal spatial structure of polymer materials consists mainly of molecular chains and free volumes. The free volume is dispersed in the material in the form of “pores”, whose size and distribution affect the ability of gas molecules to diffuse through the material. The study of the free volume allows the analysis and interpretation of the barrier properties of the material to gases [45]. The free-volume distribution of PA6 with different OMMT contents at different temperatures is shown in Figure 12. The blue area in the figure indicates the free-volume distribution of the composite. It can be seen from the figure that when 3% of filler is added, the blue area has the largest area and the highest amount, which indicates the highest amount of free volume. When the filler content increases, the blue area starts to decrease. When the temperature increases, the area of the blue region increases significantly, which also means an increase in the free volume. The free-volume distribution of 5% OMMT/PA6 material at different pressures is shown in Figure 13. When pressure reaches 60 MPa, the area and distribution of the blue region change significantly compared to the others. This also shows that the size and distribution of the free volume in the material affect the diffusion ability of gas molecules in the material; the larger and more numerous the free volume, the larger the diffusion coefficient of the material and the stronger the diffusion properties.

This indicates that the increase in temperature accelerates the movement of the molecules within the material and the gas, increasing the volume and the number of “pores” in the system and increasing the fractional free volume (FFV), thus increasing the diffusion coefficient and decreasing the barrier performance.

**Figure 14:**
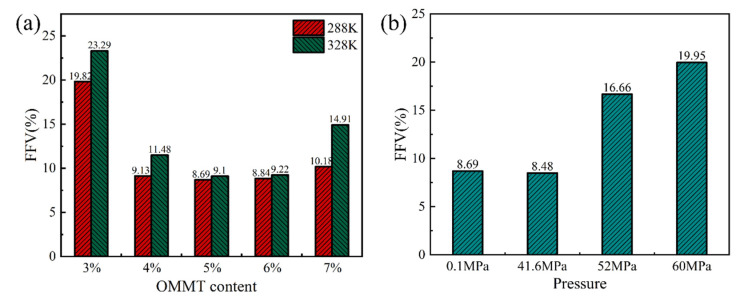
(**a**) FFV for different filler contents at different temperatures; (**b**) FFV of 5% OMMT/PA6 at four different pressure conditions.

**Figure 15:**
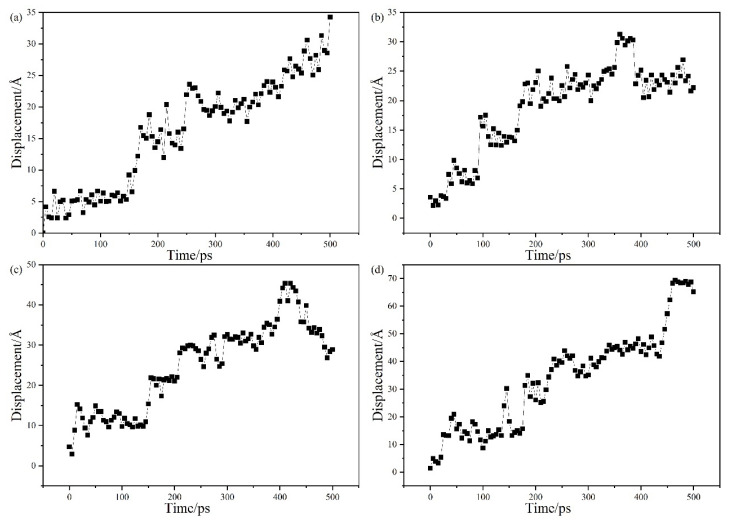
Displacement of He in 5% OMMT/PA6 at different pressures: (**a**) 0.1 MPa, (**b**) 41.6 MPa, (**c**) 52 MPa and (**d**) 60 MPa.

To further analyze the effect of the free volume, the FFV calculated from the simulation results is shown in Figure 12 and Figure 13. Figure 14a shows the FFV for different OMMT contents at different temperatures. The figure shows that the FFV is at its maximum with the addition of 3% filler. As the OMMT content increases, the FFV first decreases and then increases. The FFV reaches its lowest value when the filler content reaches 5%, which is about 50% lower than when 3% OMMT is added. However, when OMMT is increased to 6%, the FFV increases slightly. These results indicate that the increase in the OMMT limits the movement of the molecular chains inside the PA6 material, which disrupts the continuity of the “pores” in the system to some extent and reduces the FFV. As a result, the diffusion of gas molecules in the material is limited and the gas barrier properties are enhanced. When the filler is added in excess, new “pores” are created inside the material, the free volume starts to rise, gas diffusion becomes easier and the barrier performance becomes worse. In addition to this, it can be seen from the figure that temperature affects the FFV of the material. The increase in temperature accelerates the movement of the molecules inside the material and the gas, increasing the size and number of “pores” in the system. The FFV affects the diffusion of the gas, and therefore the barrier performance of the material becomes worse. Figure 14b shows the FFV of 5% OMMT/PA6 under four different pressure conditions. It can be seen from the figure that the FFV also decreases slightly when pressure increases to 41.6 MPa. This takes place because pressure makes the distance between molecules in the composite shorter, resulting in a slight decrease in the size and number of pores inside the system. When pressure increases to 50 MPa, the FFV expands rapidly. The increase in pressure forces the material molecules to move more vigorously in the system. The diffusion trajectory of helium at different pressures is shown in Figure 15. It can be seen from the figure that helium achieves jump diffusion during the simulation. Excessive pressure leads to a rapid increase in the FFV, which affects the internal “pores” of the material and increases the number of helium molecules jumping diffusion within the system. The significant increase in the displacement of helium molecules also indicates an increase in the probability of the gas passing through the “pores”, which facilitates the diffusion of the gas. At the same time, pressure accelerates the migration of the gas molecules inside the material, which also facilitates their diffusion and penetration.

## 4. Conclusions

In this paper, the barrier properties of OMMT/PA6 composites at different filler contents, different temperatures and different pressures were investigated using molecular dynamics methods. The results show that the increase in the OMMT content in PA6 can effectively limit the diffusion movement of the gas molecules within the material, thus further improving the barrier properties. When the content of OMMT added reached 5%, the permeability coefficient of the material was lower than 2 × 10^−13^ cm^3^∙cm/(cm^2^∙s∙Pa). When more OMMT was added, the permeability coefficient increased instead. This indicates that 5% OMMT content in PA6 is most suitable for the best barrier performance. Temperature also has an effect on the barrier properties of the material. The increase in temperature caused a decrease in the adsorption capacity of the material, a significant increase in diffusion capacity and a deterioration in the barrier properties. However, the permeability of the system with 5% OMMT content added remained the best for the temperature change. In addition, the barrier performance of the system with 5% OMMT content at different pressures was also investigated. It was found that the permeability coefficient decreased and the barrier properties increased when pressure reached 41.6 MPa. With the further increase in pressure, the gas diffusion coefficient increased and the barrier performance of the material decreased. Additionally, this study also found that the FFV affects the diffusion coefficient of the material. The larger the FFV, the larger the diffusion coefficient of the material.

## Figures and Tables

**Table 1:** Coordinates, charges and Lennard-Jones parameters of MMT atoms and SPC/E water in the initial model.

Atom	X/Å	Y/Å	Z/Å	$q\;\left(e\right)$	$\sigma_{i\;}\left(Å\right)$	$\varepsilon_{i}\;\left(kcal/mol\right)$
Al	0.000	3.020	12.500	3.000	0.000	0.000/13.192
Si	0.472	1.510	9.580	1.200	1.840	13.192
O(a)	0.122	0.000	9.040	−1.000	3.166	0.653
O(o)	−0.686	2.615	9.240	−1.424	3.166	0.653
O(t)	0.772	1.510	11.200	−0.800	3.166	0.653
O(OH)	0.808	4.530	11.250	−0.848	3.166	0.653
H(OH)	−0.103	4.530	10.812	0.424	0.000	0.000
Na^+^	0.000	4.530	6.250	1.000	2.586	0.418

(a) apical, (o) octahedral, (t) tetrahedral.

**Table 2:** Solubility, diffusion coefficient and permeation coefficient of helium in different systems.

Material System	Solubility $\left(cm^{3}\cdot cm^{-3}\cdot Pa^{-1}\right)$	Diffusion Coefficient $\left(cm^{2}\cdot s^{-1}\right)$	Permeation Coefficient $\left(cm^{3}\cdot cm/\left(cm^{2}\cdot s\cdot Pa\right)\right)$
3% OMMT/PA6	$3.23\times 10^{-7}$	$1.72\times 10^{-6}$	$5.56\times 10^{-13}$
4% OMMT/PA6	$1.93\times 10^{-7}$	$1.65\times 10^{-6}$	$3.18\times 10^{-13}$
5% OMMT/PA6	$1.26\times 10^{-7}$	$1.48\times 10^{-6}$	$1.85\times 10^{-13}$
6% OMMT/PA6	$1.55\times 10^{-7}$	$1.58\times 10^{-6}$	$2.45\times 10^{-13}$
7% OMMT/PA6	$1.60\times 10^{-7}$	$2.21\times 10^{-6}$	$3.54\times 10^{-13}$

**Table 3:** Solubility, diffusion coefficient and permeation coefficient of helium in different systems at 328 K.

Material System	Solubility $\left(cm^{3}\cdot cm^{-3}\cdot Pa^{-1}\right)$	Diffusion Coefficient $\left(cm^{2}\cdot s^{-1}\right)$	Permeation Coefficient $\left(cm^{3}\cdot cm/\left(cm^{2}\cdot s\cdot Pa\right)\right)$
3% OMMT/PA6	$2.58\times 10^{-7}$	$5.38\times 10^{-6}$	$1.39\times 10^{-12}$
4% OMMT/PA6	$1.51\times 10^{-7}$	$3.58\times 10^{-6}$	$5.41\times 10^{-13}$
5% OMMT/PA6	$1.02\times 10^{-7}$	$2.66\times 10^{-6}$	$2.71\times 10^{-13}$
6% OMMT/PA6	$1.17\times 10^{-7}$	$3.15\times 10^{-6}$	$3.63\times 10^{-13}$
7% OMMT/PA6	$1.32\times 10^{-7}$	$4.87\times 10^{-6}$	$6.43\times 10^{-13}$

**Table 4:** Solubility, diffusion coefficient and permeation coefficient for PA6 with 5% OMMT content of helium at different pressures.

Material System	Solubility $\left(cm^{3}\cdot cm^{-3}\cdot Pa^{-1}\right)$	Diffusion Coefficient $\left(cm^{2}\cdot s^{-1}\right)$	Permeation Coefficient $\left(cm^{3}\cdot cm/\left(cm^{2}\cdot s\cdot Pa\right)\right)$
0.1 MPa	$1.26\times 10^{-7}$	$1.48\times 10^{-6}$	$1.86\times 10^{-13}$
41.6 MPa	$1.26\times 10^{-7}$	$1.38\times 10^{-6}$	$1.74\times 10^{-13}$
52 MPa	$1.26\times 10^{-7}$	$3.41\times 10^{-6}$	$4.30\times 10^{-13}$
60 MPa	$1.26\times 10^{-7}$	$4.09\times 10^{-6}$	$5.15\times 10^{-13}$

## Data Availability

Not applicable.

## Sample Availability

Samples of the compounds are not available from the authors.
